# Relationship Between Risk of Hyper-Low-density Lipoprotein Cholesterolemia and Evacuation After the Great East Japan Earthquake

**DOI:** 10.2188/jea.JE20200267

**Published:** 2022-06-05

**Authors:** Hiroaki Satoh, Kanako Okazaki, Tetsuya Ohira, Akira Sakai, Mitsuaki Hosoya, Seiji Yasumura, Yukihiko Kawasaki, Koichi Hashimoto, Akira Ohtsuru, Atsushi Takahashi, Kazuyuki Watanabe, Michio Shimabukuro, Junichiro James Kazama, Shigeatsu Hashimoto, Gen Kobashi, Hiromasa Ohira, Hitoshi Ohto, Kenji Kamiya

**Affiliations:** 1 Department of Metabolism and Endocrinology, Juntendo University Graduate School of Medicine, Tokyo, Japan; 2 Radiation Medical Science Center for the Fukushima Health Management Survey, Fukushima Medical University, Fukushima, Japan; 3 Department of Epidemiology, Fukushima Medical University, Fukushima, Japan; 4 Department of Radiation Life Sciences, Fukushima Medical University, Fukushima, Japan; 5 Department of Pediatrics, Fukushima Medical University, Fukushima, Japan; 6 Department of Public Health, Fukushima Medical University, Fukushima, Japan; 7 Department of Pediatrics, Sapporo Medical University, Sapporo, Japan; 8 Department of Radiation Health Management, Fukushima Medical University, Fukushima, Japan; 9 Department of Gastroenterology, Fukushima Medical University, Fukushima, Japan; 10 Department of Orthopedic Surgery, Fukushima Medical University, Fukushima, Japan; 11 Department of Diabetes, Endocrinology, and Metabolism, Fukushima Medical University, Fukushima, Japan; 12 Department of Neurology, Fukushima Medical University, Fukushima, Japan; 13 Department of Metabolism, Diabetes and Nephrology, Aizu Medical Center, Fukushima Medical University, Fukushima, Japan; 14 Department of Public Health, Dokkyo Medical University, Tochigi, Japan; 15 Research Institute for Radiation Biology and Medicine, Hiroshima University, Hiroshima, Japan

**Keywords:** evacuee, the Great East Japan Earthquake, Fukushima Health Management Survey, hyper-LDL cholesterolemia, life-style

## Abstract

**Background:**

The Great East Japan Earthquake and the Fukushima Daiichi nuclear disaster forced the evacuation of residents and led to many changes in lifestyle for the evacuees. The Comprehensive Health Check was implemented to support the prevention of lifestyle-related disease and we analyzed the effect of prolonged evacuation (average of 3.0 years) on the new onset of hyper-LDL cholesterolemia.

**Methods:**

The study participants were Japanese adults living near the Fukushima Daiichi nuclear power plant in Fukushima Prefecture. Annual health checkups focusing on metabolic syndromes were conducted for persons ≥40 years by the Specific Health Checkup. Based on data from annual checkups from 2011 or 2012, we followed 18,670 participants without hyper-LDL cholesterolemia who underwent at least one other annual checkup during 2013–2015.

**Results:**

We found that the new onset of hyper-LDL cholesterolemia was 31% higher in evacuees than in non-evacuees. Evacuees had a significantly higher prevalence of obesity, hypertension, and diabetes, and higher frequency of weight change. Furthermore, logistic regression model analysis showed that the evacuation was significantly associated with the new onset of hyper-LDL cholesterolemia after adjusting age, gender, body mass index, smoking habit, alcohol consumption, diabetes, weight change, sleep deprivation, and exercise.

**Conclusion:**

The findings of the present study suggest that prolonged evacuation after a disaster is a risk factor for the new onset of hyper-LDL cholesterolemia, and lead to an increase in cardiovascular disease. It is therefore important to follow-up evacuees and recommend lifestyle changes where necessary.

## INTRODUCTION

On March 11, 2011, the Great East Japan Earthquake (Magnitude 9.0) occurred, followed by a subsequent tsunami that killed many people and destroyed numerous homes. The Fukushima Daiichi Nuclear Power Plant of Tokyo Electric Power Company also suffered extensive damage, and the chain of events caused radiation leaks into areas along the east coast of Fukushima Prefecture. Survivors living in the area faced overwhelming stress from experiencing a major earthquake, tsunami and radioactive fallout in a span of a few weeks. In addition, radioactive contamination has long been a public health concern. The Japanese government has designated areas within a radius of 30 km from the plant as areas with high radioactivity concentration. People living in these area (approximately 146,000 evacuees) were forced to move to another area until further notice.

The Fukushima Prefectural Government has stared the Fukushima Health Management Survey (FHMS) to investigate the effects of long-term low-dose radiation exposure among survivors of the Great East Japan Earthquake.^1^ We continue to carry out the comprehensive health check for the prevention of lifestyle-related diseases among individuals living in the vicinity of Fukushima Daiichi Nuclear Power Plant in Fukushima Prefecture.^1^ Based on data from the comprehensive health check, we previously reported that evacuation is a cause of weight gain,^2^ diabetes,^3^^,^^4^ hypo-high-density lipoprotein (hypo-HDL) cholesterolemia,^5^ hypertension,^6^ metabolic syndrome,^7^ and renal dysfunction.^8^^,^^9^ Those results suggested that evacuation may be a risk factor for the development of various disorders, especially cardiovascular disease (CVD). To prevent CVD, it is important to manage dyslipidemia in addition to other risk factors. Prospective epidemiologic studies have shown that low-density lipoprotein (LDL) cholesterol (-C) significantly predicts CVD in incidental atherosclerosis, and LDL-C lowering therapy reduces the risk of CVD.^10^

Therefore, using data from annual health checkups, we conducted a longitudinal analysis to investigate the effects of prolonged evacuation on new onset hyper-LDL cholesterolemia among survivors of the Great East Japan Earthquake.

## METHODS

### Subject and study design

**Figure 1.**
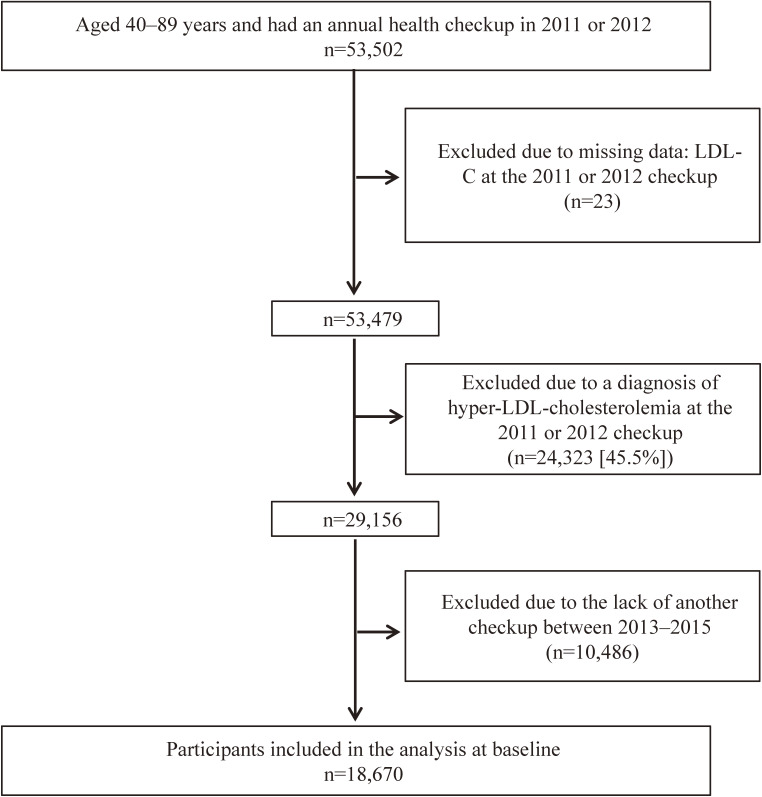
Flow diagram of the participants. Hyper-Low-density lipoprotein (LDL) cholesterolemia was defined as LDL-cholesterol level ≥140 mg/dL, or self-reported use of cholesterol-lowering drugs.

The Fukushima Health Management Survey was carried out by the Fukushima Medical University. The participants were Japanese adults living near the Fukushima Daiichi nuclear power plant in Fukushima Prefecture and residents of Tamura city, Minamisoma city, Kawamata town, Hirono town, Naraha town, Tomioka town, Kawauchi village, Okuma town, Futaba town, Namie town, Katsurao village, Iitate village, and Date city. All residents of Hirono town, Naraha town, Tomioka town, Kawauchi village, Okuma town, Futaba town, Namie town, Katsurao village, and Iitate village, and part-time residents of Tamura city, Minamisoma city, Kawamata town, and Date city were forced to evacuate their homes due to a government mandate after the disaster. Areas included in these government-designated evacuation zones were defined as the evacuation zone, and areas not included in the government-designated evacuation zones were defined as the non-evacuation zone in the present study. The participants were divided into evacuees or non-evacuees based on their residential area and not on the evacuation zone. The new shelters of the evacuees (ie, temporary house or relative house) and voluntary evacuation from the non-evacuation zone were not considered. In 2008, the Japanese government had started an annual health check program focused on detecting metabolic syndromes in adults aged 40 years or older known as “The Specific Health Check and Guidance System,” which was run by the National Health Care Insurers. In these communities, annual health checkups with a focus on metabolic syndrome for insured persons and dependents aged 40 or older by health care insurers have been conducted since 2008, with data from these annual health checkups used in the present study. We followed the dates of annual health checkups from 2011 as a part of a comprehensive health check in the Fukushima Health Management Survey. The target population of adults aged 40–89 years in these 13 municipalities was 53,502 between 2011 and 2012. We initially excluded 23 participants because of missing data such as LDL-C level as a baseline and presence of regular exercise. We also excluded 24,323 participants (45.5%) who had been diagnosed with hyper-LDL cholesterolemia at the 2011 or 2012 checkup. The final number of participants was 29,156. To conduct a longitudinal analysis, we excluded 10,486 participants who had not received another annual checkup between 2013 or 2015. Finally, 18,670 participants underwent follow-up examinations (follow-up proportion: 64.0%), and mean number of years between follow-ups was 3.0 years (Figure 1).

There were some differences in baseline characteristics between individuals who received follow-up examinations and those who did not, such as mean age (64.2 vs 60.6 years), proportion of men (50.8% vs 49.2%), evacuation (32.3% vs 25.7%), proportion of regular exercise (30.3% vs 25.8%), proportion of current smokers (15.3% vs 22.3%), proportion of excess alcohol consumption (6.9% vs 8.6%), and prevalence of hypertension (52.9% vs 48.1%), while there were few baseline differences in mean body mass index (BMI; 23.3 kg/m^2^ vs 23.4 kg/m^2^), mean LDL-C (109.6 mg/dL vs 108.4 mg/dL), prevalence of overweight participants (28.7% vs 29.4%), prevalence of diabetes (9.5% vs 9.9%), and prevalence of dyslipidemia (16.9% vs 17.6%).

### Ethics statement

Informed consent was obtained from the community representatives to conduct an epidemiological study based on guidelines of the Council for International Organizations of Medical Science.^11^ The study was approved by the Fukushima Medical University Institutional Review Board (#1319). Individuals older than 40 years were evaluated according to items in the Specific Health Examination, which is based on the Act on Assurance of Medical Care for Elderly People (Act No. 80, 1982). The participants in the Fukushima Health Management Survey provided their written informed consent at the follow-up survey. We obtained the informed consent from the participants only. To use the data before the disaster, we obtained permission from the community representatives because they have been managing the data. To compare the data before and after the disaster, we used the health checkup data managed by each community. Therefore, we obtained permission from the community representatives. As we mentioned above, we also obtained written informed consent from the participants during the follow-up survey. The participants’ consent forms are kept under lock and key in the Fukushima Health Management Survey. The consent procedure was approved by the Fukushima Medical University Institutional Review Board.

### Measurements

The examination included height, weight, abdominal circumference, BMI, blood pressure (BP), aspartate aminotransferase, alanine aminotransferase, γ-glutamyl transpeptidase, triglyceride (TG), HDL-C, low-density lipoprotein cholesterol (LDL-C), hemoglobin A1c (HbA1c), fasting plasma glucose, and urine protein and sugar level measurements. TG, HDL-C, and LDL-C before and after the disaster were measured using the Autoanalyzer JCA-BM8030 (JEOL Ltd.) at the laboratory of the Fukushima Preservative Service Association of Health, except for residents in Futaba (*n* = 758). Since the trends in TG, HDL-C, and LDL-C before and after the disaster in Futaba were essentially the same as other communities, we included the data of Futaba in the analysis. Additional measurements included in the examination were serum creatinine level, estimated glomerular filtration rate, uric acid level, urine testing for occult blood, and peripheral blood count, which includes red blood cell count, hematocrit, hemoglobin, platelet count, and white blood cell count. The value for HbA1c at the 2011 or 2012 checkup was estimated using a National Glycohemoglobin Standardization Program equivalent value calculated using the equation HbA1c (%) = 1.02 × HbA1c (JDS) (%) + 0.25%.^12^

Hyper-LDL cholesterolemia was defined as LDL-C ≥140 mg/dL, or self-reported use of lipid-lowering drugs in accordance with the JAS guidelines.^13^ Dyslipidemia in this study was defined as triglyceride level ≥150 mg/dL or HDL-C level <40 mg/dL, because the baseline excluded hyper-LDL cholesterolemia or self-reported use of lipid-lowering drugs. Obesity was defined as BMI ≥25 kg/m^2^. Diabetes was defined as a fasting plasma glucose level ≥126 mg/dL (7.0 mmol/L), HbA1c level ≥6.5%, or self-reported use of glucose-lowering drugs in accordance with the Japan Diabetes Society (JDS) guidelines.^14^ Hypertension was defined as a systolic BP ≥140 mm Hg, a diastolic BP ≥90 mm Hg, or use of blood pressure-lowering drugs.

### Statistical analysis

Data are presented as mean and standard deviation (SD). The participants were divided into two groups based on residence status after the Great East Japan Earthquake: evacuees (*n* = 6,025) and non-evacuees (*n* = 12,645). The baseline characteristics of the participants who received follow-up health examinations were compared between evacuees and non-evacuees using a chi-square test, non-paired *t*-test, or Wilcoxon rank-sum test. To evaluate the impact of evacuation on the new onset of hyper-LDL cholesterolemia, odds ratios (ORs) of new onset hyper-LDL cholesterolemia and 95% confidence intervals (CIs) for evacuees were calculated using logistic regression model analysis with model 1 and 2. Model 1 adjusted for age (continuous variables), gender, BMI (lean: less than 18.5 kg/m^2^, normal: more than 18.5 kg/m^2^ and less than 25 kg/m^2^, obesity: more than 25 kg/m^2^), current smoker, excess alcohol consumption (44 g/day or more alcohol intake), and diabetes; and model 2 adjusted for the variables in model 1, as well as weight change (≥10 kg) since age 20 years, weight change (≥3 kg) within 1 year, sleep quality (sleep provides rest or not), and regular exercise (exercise at least twice a week). We used dummy variables for missing data in the multivariable-adjusted models. SAS version 9.4 (SAS Institute, Cary, NC, USA) was used for all analyses. All probability values for statistical tests were 2-tailed, and *P* values <0.05 were considered statistically significant.

## RESULTS

### Baseline characteristics of evacuees and non-evacuees who received follow-up health examinations after the disaster

We followed 18,670 participants (9,489 men and 9,181 women) without hyper-LDL cholesterolemia based on data from annual checkups in 2011 or 2012 and those who underwent at least one other annual checkup in 2013 or 2015 (Figure 1). Table 1 shows the clinical characteristics of the two groups (non-evacuees or evacuees) at baseline. First, we compared risk factors for the development of hyper-LDL cholesterolemia. The evacuees were significantly younger (mean age 63.6; SD, 11.1 years vs 64.5; SD, 10.4 years, *P* < 0.001) and less frequently men (49.3% vs 51.5%, *P* = 0.004) than the non-evacuees. The evacuees had significantly higher BMI (23.8; SD, 3.5 kg/m^2^ vs 23.1; SD, 3.3 kg/m^2^, *P* < 0.001), higher waist circumference (84.1; SD, 9.4 cm vs 82.6; SD, 9.1 cm, *P* < 0.001), higher triglycerides levels (113.9; SD, 91.6 mg/dL vs 107.2; SD, 79.2 mg/L, *P* < 0.001), lower HDL-C levels (59.3; SD, 15.9 mg/dL vs 60.6; SD, 15.8 mg/L, *P* < 0.001), higher prevalence of obesity (34.8% vs 25.8%, *P* < 0.001), diabetes (10.8% vs 8.9%, *P* < 0.001), dyslipidemia (20.4% vs 16.0%, *P* < 0.001), and use of glucose-lowering drugs (6.6% vs 5.7%, *P* = 0.019) and blood pressure-lowering drugs (38.8% vs 36.9%, *P* = 0.019). In addition, the evacuees had significantly higher frequency of weight change (≥10 kg) since age 20 years (37.4% vs 29.0%, *P* < 0.001), weight change (≥3 kg) within 1 year (40.5% vs 20.2%, *P* < 0.001), and smoking habit (17.4% vs 14.3%, *P* < 0.001) than non-evacuees. The evacuees had a significantly lower frequency of adequate sleep (63.7% vs 73.9%, *P* < 0. 001) than non-evacuees. On the other hand, there was no significant difference in the frequency of regular exercise between evacuees and non-evacuees (30.2% vs 30.3%, *P* = 0.938). Interestingly, annual change of LDL-C levels was significantly higher in evacuees than non-evacuees (3.0; SD, 10.3 mg/dL/year vs 2.2; SD, 8.8 mg/dL/year, *P* < 0.001), although LDL-C levels were not different between the two groups (111.0; SD, 20.6 mg/dL vs 109.5; SD, 20.4 mg/dL, *P* = 0.157).

**Table 1.** Baseline characteristics of the evacuees and non-evacuees who received follow-up health examinations after the Great East Japan Earthquake

	Non-evacuees	Evacuees	*P* value^f^
Number	12,645	7,170	
Gender, % male	51.5%	49.3%	0.004
Age, years	64.5 (10.4)	63.6 (11.1)	<0.001
Follow-up periods, years	3.02 (0.87)	3.06 (0.93)	<0.001
Body weight, kg	57.6 (10.5)	59.4 (11.2)	<0.001
BMI, kg/m^2^	23.1 (3.3)	23.8 (3.5)	<0.001
Waist circumference, cm	82.6 (9.1)	84.1 (9.4)	<0.001
Obesity, %^a^	25.8	34.8	<0.001
Hypertension, %^b^	52.4	53.9	0.063
Blood pressure-lowering drugs, %	36.9	38.8	0.009
Diabetes, %^c^	8.9	10.8	<0.001
Glucose-lowering drugs, %	5.7	6.6	0.019
Triglycerides, mg/dL	107.2 (79.2)	113.9 (91.6)	<0.001
HDL-C, mg/dL	60.6 (15.8)	59.3 (15.9)	<0.001
LDL-C, mg/dL	109.5 (20.4)	111.0 (20.6)	0.157
Annual change of LDL-C, mg/dL/year	2.2 (8.8)	3.0 (10.3)	<0.001
Dyslipidemia, %^d^	16.0	20.4	<0.001
Weight change, ≥10 kg from age 20 years, %	29.0	37.4	<0.001
Weight change, ≥3 kg within 1 year, %	20.2	40.5	<0.001
Regular exercise, %	30.3	30.2	0.938
Adequate sleep, %	73.9	63.7	<0.001
Current smoker, %	14.3	17.4	<00001
Alcohol consumption, %			<0.001
Non-drinker	49.5	50.4	
Light drinker	44.1	41.6	
Moderate/heavy drinker^e^	6.4	8.0	

Data are presented as mean (standard deviation).

Data are mean values (standard deviation) for continuous variables and percentage values for categorical variables, unless otherwise noted.

^a^Obesity was defined as body mass index ≥25 kg/m^2^.

^b^Hypertension was defined as systolic blood pressure ≥140 mm Hg, diastolic blood pressure ≥90 mm Hg or self-reported use of blood pressure-lowering drugs.

^c^Diabetes was defined as fasting glucose level ≥126 mg/dL, Hemoglobin A1c level ≥6.5%, or self-reported use of glucose-lowering drugs.

^d^Dyslipidemia was defined as triglyceride level ≥150 mg/dL, or HDL-C level <40 mg/dL, because the baseline excluded hyper-LDL cholesterolemia.

^e^Moderate/heavy drinker was defined as ethanol intake ≥44 g/day.

^f^*P* values comparing the evacuee to the non-evacuee group after the earthquake used the chi-square test, non-paired *t*-test.

### Risk factors for the new onset of hyper-LDL cholesterolemia after disaster

**Figure 2.**
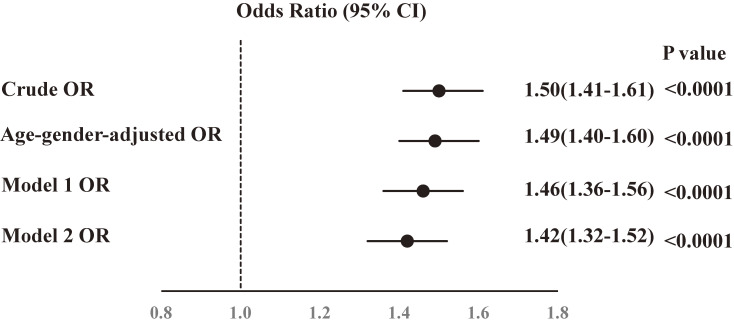
Odds ratios (ORs) and 95% confidence intervals (CIs) of (b) new-onset hyper low-density lipoprotein (LDL) cholesterolemia in evacuees after the Great East Japan Earthquake. Model 1 OR: adjusted for age (continuous variables), gender, body mass index (lean, normal, obesity), current smoker, excess ethanol intake, diabetes using logistic regression model analysis. Model 2 OR: adjusted for age (continuous variables), gender, body mass index (lean, normal, obesity), current smoker, excess ethanol intake, diabetes, weight change (≥10 kg) from age 20 years, weight change (≥3 kg) within 1 year, sleep quality, and regular exercise using logistic regression model analysis.

Next, we investigated the new onset of hyper-LDL cholesterolemia. The cumulative number of participants with new onset hyper-LDL cholesterolemia was 28.0% (5,231 participants) over a mean follow-up period of 3.0 years. The new onset rate of hyper-LDL cholesterolemia was 92.4/1,000 person-years, and that of the evacuees was 1.32-fold higher (110.4/1,000-person-years) than that of non-evacuees (83.7/1,000-person-years). Significant differences were observed in baseline characteristics between the two groups, which is why we applied logistic models. ORs of the risk factors for the development of hyper-LDL cholesterolemia are shown in Figure 2. Evacuation was a significant risk factor for the development of hyper-LDL cholesterolemia compared with non-evacuation, with crude OR of 1.50 (95% CI, 1.41–1.61). The age-gender-adjusted OR for evacuation was 1.49 (95% CI, 1.40–1.60). After adjusting for age, gender, BMI, smoking, alcohol consumption, and diabetes, the multivariate OR for evacuation was 1.46 (95% CI, 1.36–1.56). Furthermore, after adjusting for weight change (≥10 kg) since age 20 years, weight change (≥3 kg) within 1 year, sleep quality, and regular exercise, and adding to age, gender, BMI, smoking, alcohol consumption, and diabetes, the multivariate OR for evacuation was 1.42 (95% CI, 1.32–1.52). In addition, because there are missing data for lifestyle factors such as regular exercise (*n* = 8,287), change in weight since age 20 years (*n* = 8,285), change in weight within 1 year (*n* = 8,284), and restorative sleep (*n* = 8,251), we reanalyzed 10,331 participants, which did not include those with missing data for lifestyle factors. As a result, the associations were basically unchanged. After adjusting for age, gender, BMI, smoking, alcohol consumption, and diabetes, the multivariate OR for evacuation was 1.51 (95% CI, 1.38–1.65). Furthermore, after adjusting weight change (≥10 kg) since age 20 years, weight change (≥3 kg) within 1 year, sleep quality, and regular exercise, adding to age, gender, BMI, smoking, alcohol consumption, and diabetes, the multivariate OR for evacuation was 1.48 (95% CI, 1.35–1.62).

## DISCUSSION

The main findings of the present study were that new onset of hyper-LDL cholesterolemia was significantly higher in evacuees than non-evacuees after the Great East Japan Earthquake and Fukushima Daiichi nuclear disaster. Interestingly, annual change of LDL-C levels was significantly higher in evacuees than non-evacuees, although baseline LDL-C levels were not different between the two groups. Furthermore, we investigated the effect of evacuation on the new onset of hyper-LDL cholesterolemia after adjusting for baseline lifestyle factors such as weight change, regular exercise, and sleep quality. The results revealed that evacuation after the Great East Japan Earthquake and Fukushima Daiichi nuclear disaster was an independent risk factor for the new onset of hyper-LDL cholesterolemia, even after adjusting various lifestyle factors. However, because the evacuees had a significantly higher frequency of weight change (≥3 kg) within 1 year and smoking habit, and lower frequency of adequate sleep than non-evacuees, long-term changes in lifestyle may induce the new onset of hyper-LDL cholesterolemia. Therefore, continuous surveys should be performed for the evacuees for long-term monitoring. Substantial epidemiological studies have demonstrated that LDL-C level is a strong and independent positive risk factor for development of CVD.^10^ Therefore, evacuation may lead to an increase in the incidence of CVD. However, LDL-C lowering therapy has been repeatedly demonstrated to reduce CVD risk in many populations.^10^ Since hyper-LDL cholesterolemia is a well-established but modifiable CVD risk factor, it is very important to conduct periodic health checkups and give lifestyle recommendations for evacuees.

The mechanisms by which evacuation increases the incidence of new onset hyper-LDL cholesterolemia are not fully understood, but two possibilities may explain the association between evacuation and increased LDL-C levels. First, living as an evacuee increases stress in terms of privacy, availability of food, duty assignments, income, jobs, and health.^15^ Our FHMS group has reported that the evacuees tended have a lower intake of vegetables and soy-beans and a higher intake of processed juices as compared with the non-evacuees after the disaster.^15^^–^^17^ These reports suggest an increase in health-damaging behaviors, such as unhealthy diets and sedentary lifestyle. High intake of juices combined with lack of regular exercise are major causes of abdominal obesity, and increased visceral obesity increases free fatty acids in the portal vein, promotes lipoprotein synthesis, and leads to increased TG and LDL-C levels as well as decreased HDL-C levels. Indeed, our data show that evacuees had adverse metabolic factors including obesity, diabetes, and dyslipidemia. In addition, the ratios of weight change (≥10 kg) from age 20 years, weight change (≥3 kg) within 1 year, and sleep deprivation, which were all significantly higher compared to non-evacuees. These results suggest that the evacuees may have experienced a change in their lifestyle more than non-evacuees after the disaster. Second, many epidemiological studies have revealed that the excessive intake of cholesterol and animal fat results in increased serum LDL-C levels.^10^ Evacuees may have changed their diet after the disaster to one which was high in cholesterol. Because excessive intake of cholesterol not only causes the development of hyper-LDL cholesterolemia but also causes abdominal obesity, this may lead to an increase in TG and decrease in HDL-C levels, which are consistent with our results. Although the increase in new onset hyper-LDL cholesterolemia after disasters may be affected by numerous factors such as a change in diet, reduction in exercise, and psychological stress; the association between evacuation and new onset hyper-LDL cholesterolemia remains unclear. We have previously reported that evacuation is a cause of weight gain,^2^ diabetes,^3^^,^^4^ hypo-HDL cholesterolemia,^5^ hypertension,^6^ metabolic syndrome,^7^ and renal dysfunction.^8^^,^^9^ Most of these reports have so far investigated the short-term effects before and after the disaster and does not include the long-term effects over the years following the disaster. Therefore, the strength of this study is that we investigated the long-term effects following evacuation.

Several limitations are present in this study. First, we did not evaluate the period of evacuation. We did not have information on whether they are still being evacuated or not. The period of evacuation varied depending on the individual, but all residents of the evacuation zone have been evacuated until at least the end of March 2012. However, once evacuated, it is important that evacuation is a risk factor for the development of hyper-LDL cholesterolemia. The results of this study may underestimate the effects of evacuation but are unlikely to overestimate them. Second, we did not conduct follow-up to residents who received no annual checkup or only one annual checkup. There were some differences in baseline characteristics between individuals who received follow-up examinations and those who did not such as mean age, proportion of men, evacuation, and proportion of regular exercise, proportion of current smoker, proportion of excess alcohol consumption, and prevalence of hypertension, while there were few baseline differences in mean BMI, mean LDL-C, prevalence of overweight participants, prevalence of diabetes, and prevalence of dyslipidemia. Therefore, this selection bias may have affected our results. Third, analyzed data in the present study indicated that there is a decline in the frequency at which residents receive health checks over time. These findings appear to indicate a decrease in interest in the health checks provided by the FHMS. This decline could affect the results of the survey, so it is necessary to raise interest in these health checks through advertising and better education. Fourth, we did not assess the impact of lifestyle changes during the follow-up period because we investigated the impact of baseline lifestyle factors on new onset hyper-LDL cholesterolemia.

Therefore, it could not be determined whether the continuation of lifestyle factors affected the development of hyper-LDL cholesterolemia. Finally, we used baseline clinical characteristics and evacuation as confounders in our multivariate analysis. We cannot completely rule out the possibility of residual confounding, such as that by socioeconomic factors, before the disaster. These socioeconomic factors may have influenced the association between evacuation and new onset hyper-LDL cholesterolemia.

In conclusion, the findings of the present study suggest that prolonged evacuation after a disaster is a risk factor for the development of hyper-LDL cholesterolemia, leading to an increase in incidence of CVD. This information could prove important for the need for periodic health checkups and lifestyle recommendations for evacuees in the future.
